# Supplementary material for: Neural computations underlying inverse reinforcement learning in the human brain
Source: eLife. 2017 Oct 30;6:e29718. doi: 10.7554/eLife.29718 (PMC5662289; doi:10.7554/eLife.29718)
Supplement: Figure 3—source data 1. — OFC: orbitofrontal cortex, dmPFC: dorsomedial prefrontal cortex. x y z in MNI coordinates. [file elife-29718-fig3-data1.docx]

| **map** | **Regions** | **x** | **y** | **z** | **Voxel number at p < 0.001**  **cluster FWE** | **T-score** |
| --- | --- | --- | --- | --- | --- | --- |
|  |  |  |  |  |  |  |
|  |  |  |  |  |  |  |
|  |  |  |  |  |  |  |
|  |  |  |  |  |  |  |
| predicted outcome  (sim + dis) | occipital lobe | 44 | -74 | -10 | 3540 | 4.80 |
|  | left OFC | -42 | 46 | -4 | 383 | 4.31 |
|  | dmPFC | 0 | 40 | 40 | 712 | 5.14 |

**TABLE S2 – related to Figure 3:** areas exhibiting significant changes in BOLD associated with predicted outcome in similar and dissimilar. OFC: orbitofrontal cortex, dmPFC: dorsomedial prefrontal cortex. x y z in MNI coordinates.
