# Supplementary material for: Neural computations underlying inverse reinforcement learning in the human brain
Source: eLife. 2017 Oct 30;6:e29718. doi: 10.7554/eLife.29718 (PMC5662289; doi:10.7554/eLife.29718)
Supplement: Figure 4—source data 1. — Pre-SMA: pre-supplementary motor area. TPJ: temporo-parietal junction. dlPFC: dorsolateral prefrontal cortex. x y z in MNI coordinates. [file elife-29718-fig4-data1.docx]

| **map** | **regions** | **x** | **y** | **z** | **Voxel number at p < 0.001 cluster FWE** | **T-score** |
| --- | --- | --- | --- | --- | --- | --- |
|  |  |  |  |  |  |  |
|  |  |  |  |  |  |  |
|  |  |  |  |  |  |  |
|  |  |  |  |  |  |  |
| learning signals  (sim + dis) | pre-SMA | -6 | 18 | 44 | 912 | 5.76 |
|  | striatum | -20 | 10 | 10 | 179 | 4.99 |
|  | right ant. insula | 50 | 22 | 10 | 455 | 5.49 |
|  | left ant. insula | -38 | 24 | -6 | 134 | 4.54 |
|  | TPJ | 58 | -46 | 0 | 233 | 4.63 |
|  | occipital lobe | 42 | -48 | -14 | 1515 | 6.54 |
|  | dlPFC | 44 | 40 | 26 | 538 | 5.72 |

**TABLE S3 – related to Figure 4:** areas exhibiting significant changes in BOLD associated with entropy signals. Pre-SMA: pre-supplementary motor area. TPJ: temporo-parietal junction. dlPFC: dorsolateral prefrontal cortex. x y z in MNI coordinates.
